# Editorial: Functional genomics of secondary metabolite biosynthesis in medicinal plants

**DOI:** 10.3389/fgene.2023.1223492

**Published:** 2023-06-20

**Authors:** Monica Saifi, Mather Ali Khan, M. Z. Abdin

**Affiliations:** ^1^ Centre for Transgenic Plant Development, Department of Biotechnology, School of Chemical and Life Sciences, Jamia Hamdard, New Delhi, India; ^2^ Bond Life Sciences Center, University of Missouri, Columbia, MO, United States

**Keywords:** functional genomics, secondary metabilites, transcriptomics, medicinal and aromatic plants, genomics

Tens of thousands of secondary metabolites produced by medicinal plants are utilized in traditional medicine and are crucial for the production of medicines, food additives, and essential oils, among other products. To address the low concentrations of these phytocompounds in plants, it is of critical importance to study the regulation of the biosynthetic pathways of these phytocompounds in plants. The technologies such as whole genome sequencing when integrated with proteomics and metabolomics improved our comprehension of these biosynthetic routes.

Technology progress has been essential to the growth of plant genomics. With every new technical development, genomics has advanced significantly faster. The fundamentals in sequencing technologies were developed in the year 1970 and since then three generations of advancement have taken place. With each new day, it keeps growing and changing. The arrival of whole genome sequencing, methods to assemble them, and cutting-edge bioinformatics tools to support them has revived the field of molecular biology and genomics of medicinal plants (Alami et al., 2022). The identification of genes that contribute to the production of plant secondary metabolites and the clarification of their evolutionary history will be made possible with the aid of substantial and in-depth molecular genetics study, which will be made possible with the aid of high-quality genome sequences. Whole-genome sequencing provides a solid base for performing fundamental research on molecular breeding of medicinal plants and facilitating the development of new cultivars. Additionally, by utilizing the species’ genomic data and regulatory system in conjunction with omics technologies, genomic research on medicinal plants seeks to understand their molecular mechanisms for preventing human diseases and demonstrate their impact on the human body at the genomic level.

Genome sequence-based discovery has opened up new perspectives on the variety and evolution of medicinal plants. It also makes comparative genomics research easier and offers useful resources for researching the synthesis of pharmaceutically important phytocompounds.

The study of functional genomics has advanced due to the exponential rise of whole-genome sequence data for plants, particularly medicinal plants. Finding possible candidate genes for improving the manufacture of secondary plant metabolites requires access to transcriptomics and short RNA sequencing data sources from a variety of plant tissues. A few crucial genes for trait enhancement at the DNA, RNA, and protein levels are discovered after a genome-wide analysis of multiple important genes and transcription factors. The functional significance of putative genes, promoters, long noncoding RNA, and microRNA must be determined. To accomplish these goals, a variety of genetic engineering methods, including overexpression and RNAi, as well as genome-editing technologies, including ZNF, TALENS, and CRISPR/Cas, have been applied in a variety of plants. The CRISPR/Cas system and its offshoots have developed into a robust, effective, and adaptable tool for gene editing, transcriptional activation, and suppression, among other uses. In order to improve crucial traits in plants, such as high concentrations of desirable secondary metabolites, extraordinary flavours, higher yields, and improved nutritional and medicinal value, functional genomics and genome editing are commonly applied. We invited reviews and research publications on functional genomics and genome editing in medicinal plants for this Research Topic.

In order to increase the plant secondary metabolites production, the present Research Topic sought to publish basic and translational research across the gamut of functional genomics techniques and applications. In total, four articles on this Research Topic were published. The Research Topic addressed studies showing how functional genomics has improved secondary plant metabolites in medicinal plants.

A combined transcriptome and metabolome analysis of the tuber, stem, and leaf of *C. convolvulacea* was performed by Yuan et al. who discovered 1,144 metabolites and 231,840 unigenes in three different experimental groups. The research showed that the three tissues had a wide range of differences. Alkaloids and flavonoids were abundant in the stems and leaves, respectively, and organic acids, flavonoids, and amino acids and their derivatives were abundant in the tubers. Candidate genes implicated in the production of flavonoids, tryptophan, and alkaloids were discovered by transcriptome sequencing. Their team claimed that the expression of CHI, CYP73A, C3′H, F3H, CYP75B1, anthocyanidin synthase, and FLS is related to the variance in the isoflavone content. The expression of TDC/DDCs in the corresponding tissues was likewise consistent with the levels of tryptamine, L-tyrosine and indole. ASP5, ARO8, GOT, and AOC3 expression levels also showed that downstream metabolites were produced on metabolization of L-tryptophan. Collectively, their datasets pose a wealth of research questions and serve as a valuable resource for work to come on the applications of this medicinal plant.

*Oliveria decumbens* flower and root samples were compared transcriptomically by Khodavirdipour et al. RNA-seq was used to assess the expression levels of the genes contributing to biosynthesis of terpenoids, and the phytochemicals present in the essential oil were determined by GC/MS. Two samples of blossom and root have yielded a combined 136,031,188 reads. The outcome indicates that the root and blossom are the two parts of the plant where MEP is the most active route. Twenty three important genes were found to be involved in the biosynthesis of terpenes, and three genes of GPP, FPPS, and GGPP, which are the precursors in the production of mono, di, and triterpenes, are elevated in the root. Three genes, among them, showed the greatest upregulation in the root, while three other genes were expressed solely in the flower. In the meantime, 191 and 185 upregulated genes from the plant’s flower and root, respectively, were chosen for gene ontology analysis and co-expression network reconstruction. The *O. decumbens* transcriptome was the subject of the first study of its sort, and 67 genes that have been added to the NCBI database were discussed. Taking all the data from this study into account, new insights on *O. decumbens* Vent’s genetic makeup are revealed, paving the path for future advances in medical/plant biotechnology and the pharmaceutical business.

In conclusion, Functional Genomics provides a very promising approach for enhancement of secondary metabolites in plants. The results of functional genomics in different medicinal plants (e.g., *C. convolvulacea*, *O. decumbens*, etc.) presented in this Research Topic will provide better insight to the plant biotechnology researchers and pharmaceutical industries for the development of plant based herbal medicines using these medicinal plants.
